# Supplementary material for: Heat adaptation of phage T7 under an extended genetic code
Source: Virus Evol. 2021 Dec 1;7(2):veab100. doi: 10.1093/ve/veab100 (PMC8923235; doi:10.1093/ve/veab100)
Supplement: veab100_Supp [file veab100_supp.zip › Sequencing_Analysis.zip!/Sequencing_Analysis/A2/calibration/A2_52_S18_L001_R1_001.error_rates.pdf]

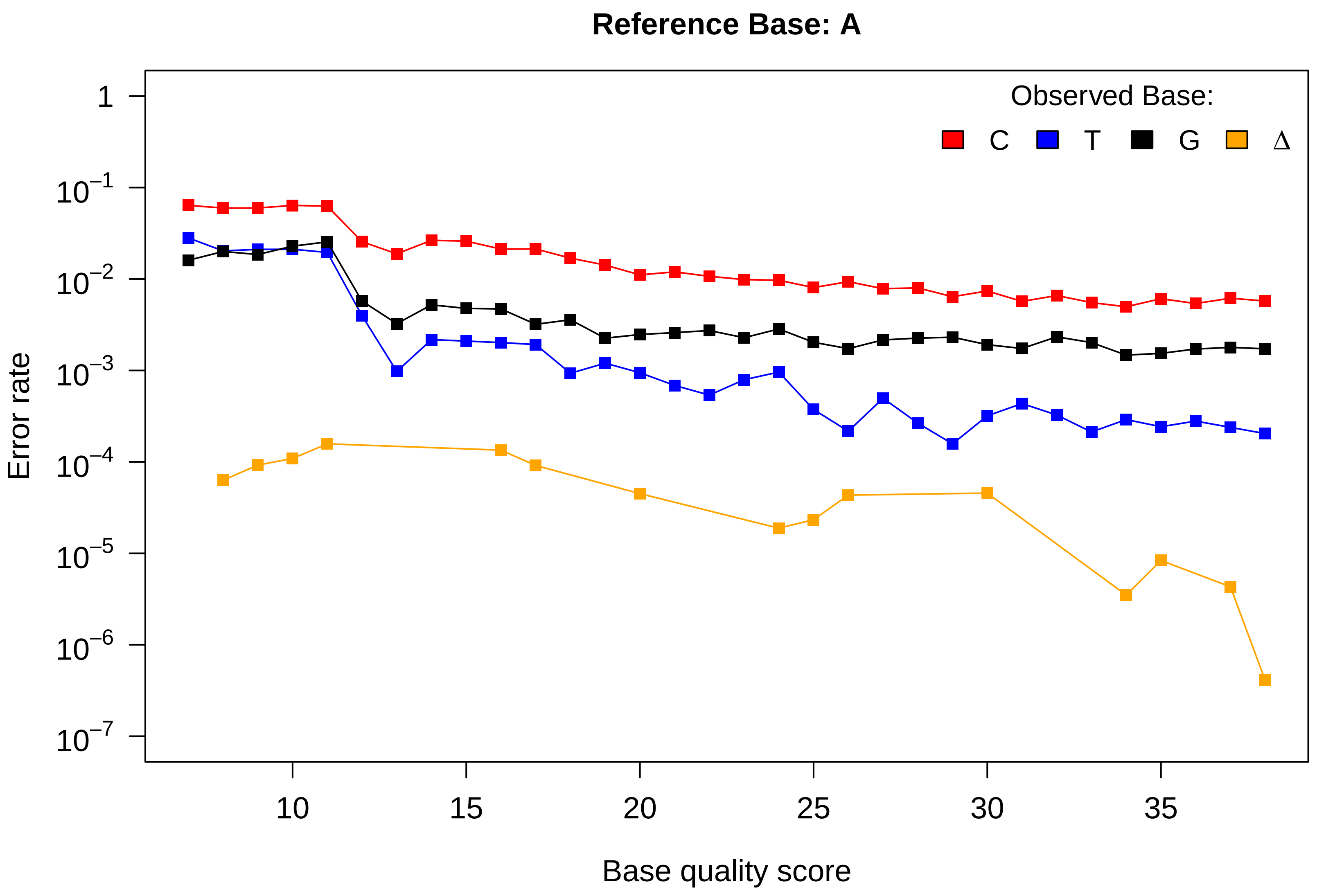

Reference Base: A
Observed Base:
C
T
G
Δ
Error rate
1
10^{-1}
10^{-2}
10^{-3}
10^{-4}
10^{-5}
10^{-6}
10^{-7}
10
15
20
25
30
35
Base quality score

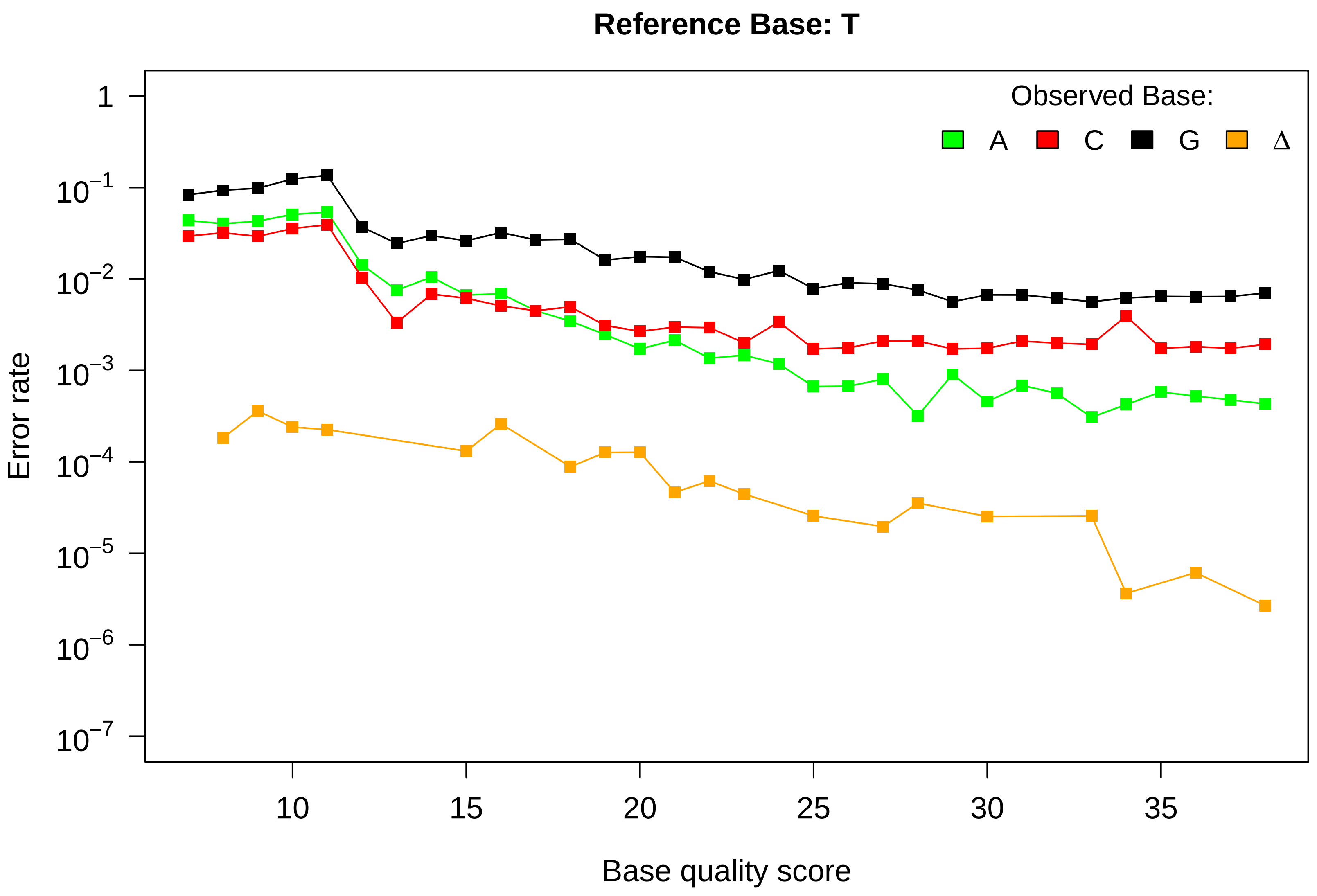
Reference Base: T
Observed Base:
A
C
G
Δ
Error rate
1
10^{-1}
10^{-2}
10^{-3}
10^{-4}
10^{-5}
10^{-6}
10^{-7}
10
15
20
25
30
35
Base quality score

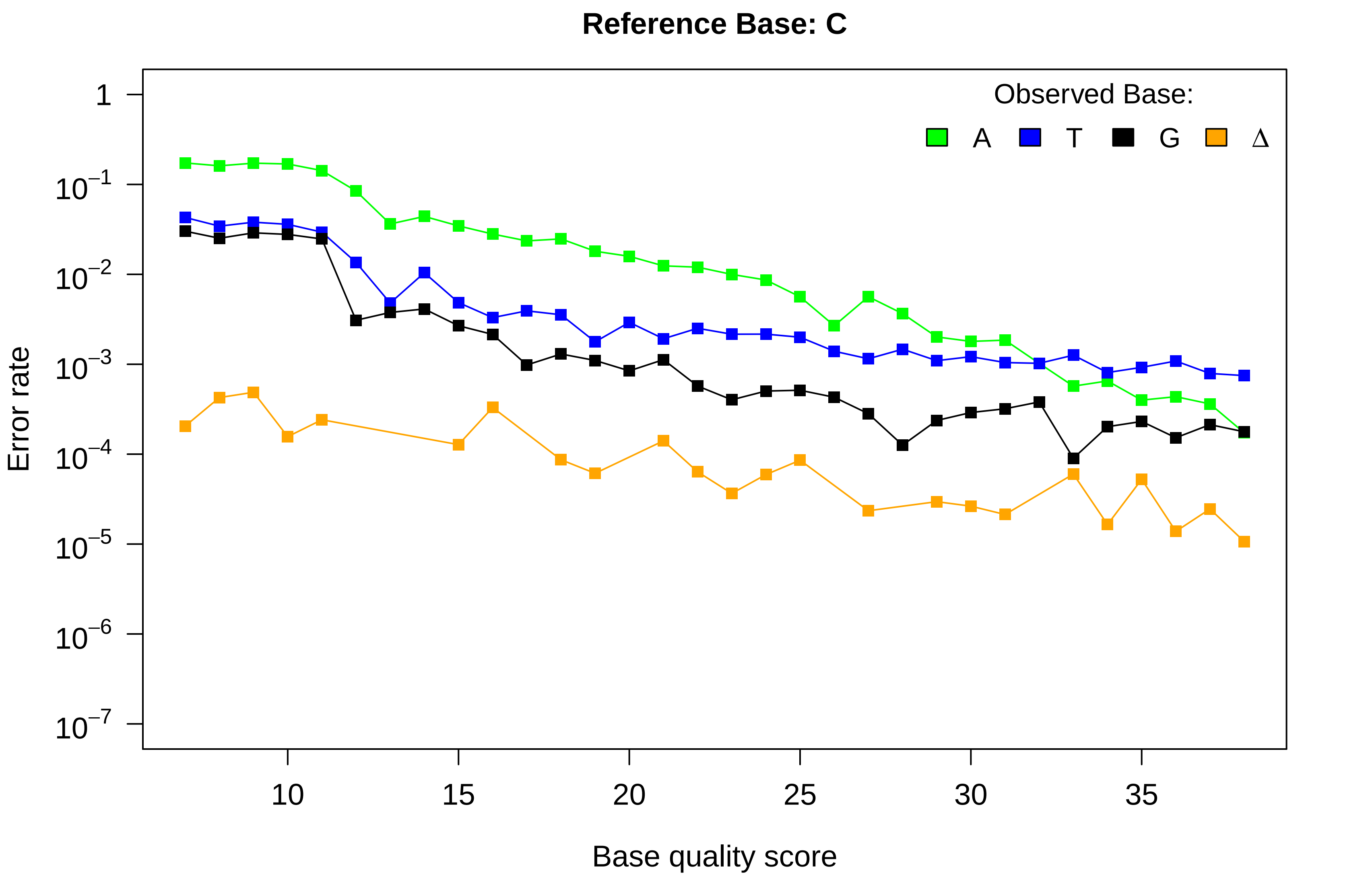
Reference Base: C
Observed Base:
A
T
G
Δ
Error rate
1
10^−1
10^−2
10^−3
10^−4
10^−5
10^−6
10^−7
10
15
20
25
30
35
Base quality score

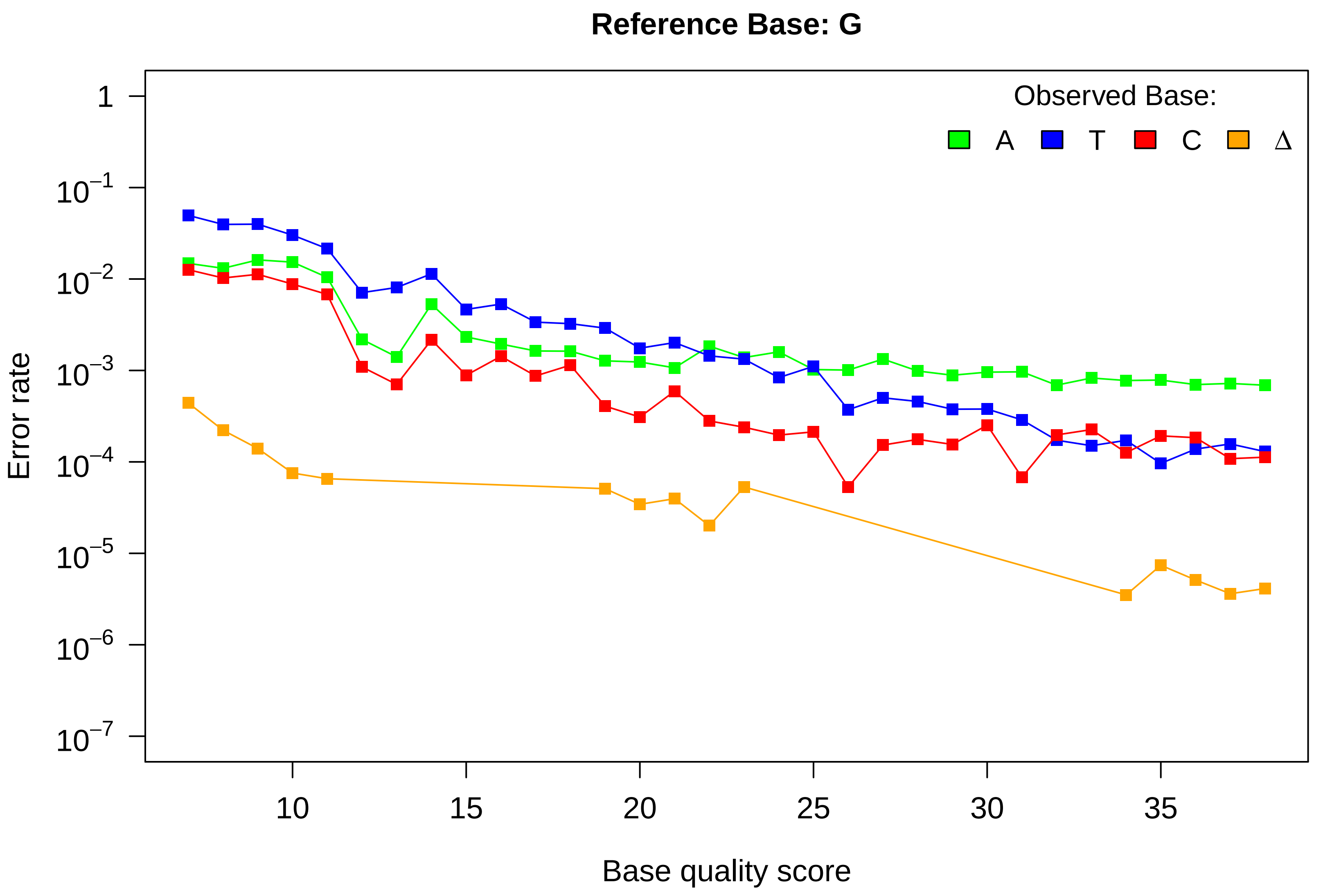
Reference Base: G
Observed Base:
A
T
C
Δ
1
10−1
10−2
10−3
10−4
10−5
10−6
10−7
Error rate
10
15
20
25
30
35
Base quality score

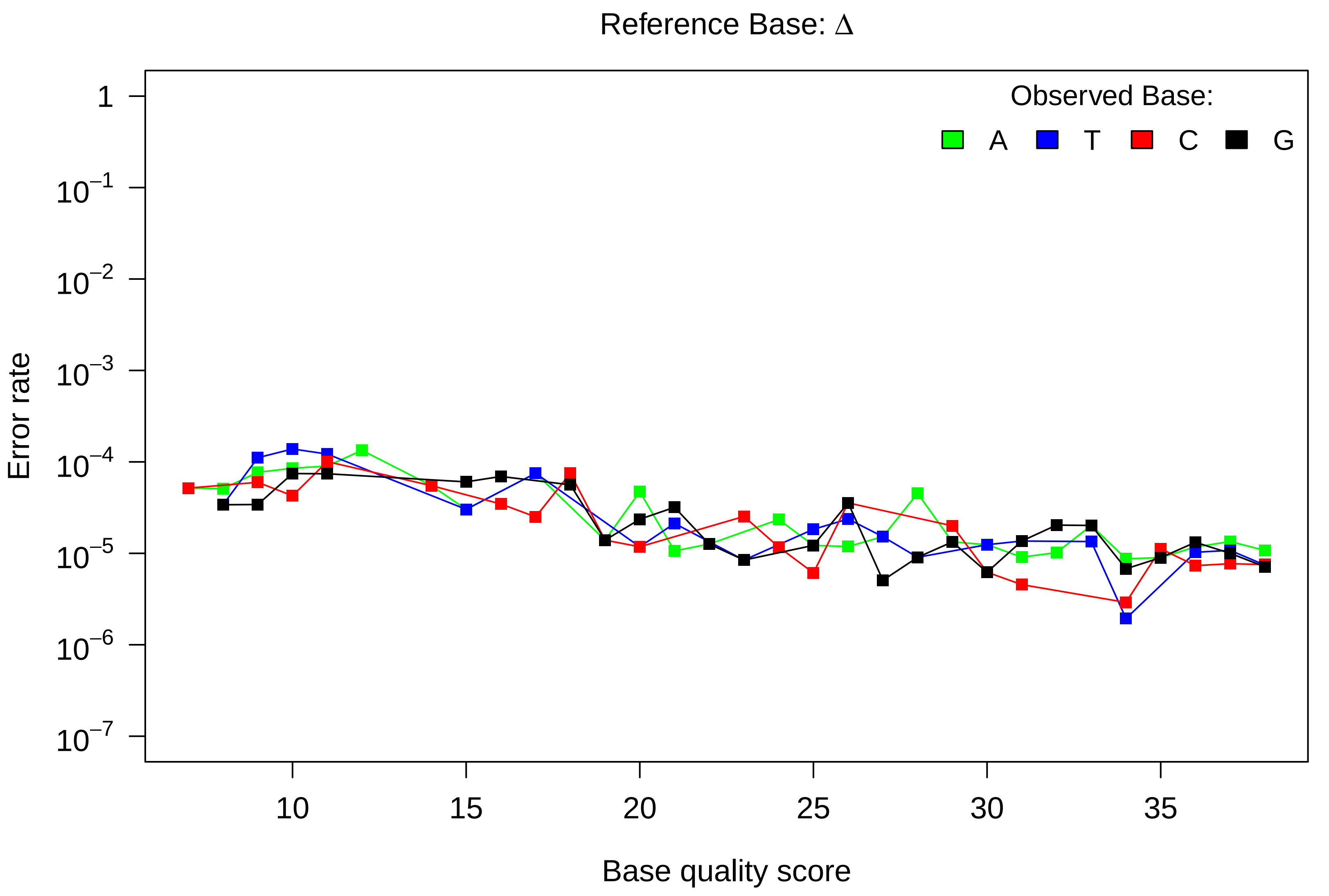

Reference Base: Δ
Observed Base:
A
T
C
G
Error rate
1
10^−1
10^−2
10^−3
10^−4
10^−5
10^−6
10^−7
10
15
20
25
30
35
Base quality score
